# Female sexual function after transobturator tape in women with urodynamic stress urinary incontinence

**DOI:** 10.1186/2193-1801-3-570

**Published:** 2014-09-30

**Authors:** Abdulmuttalip Simsek, Faruk Ozgor, Bahar Yuksel, Onur Kucuktopcu, Sinan Levent Kirecci, Mehmet Toptas, Omer Sarılar, Ahmet Yalcin Berberoglu, Zafer Gokhan Gurbuz, Saban Mimaroglu, Fatih Akbulut, Murat Baykal, Burak Arslan, Metin Savun, Burak Ucpinar

**Affiliations:** Department of Urology, Haseki Research and Training Hospital, Millet Cad. No: 11, Fatih, Istanbul 34098 Turkey; Department of Urology, Sisli Etfal Research and Training Hospital, Istanbul, Turkey; Department of Obstetrics and Gynecology, Istanbul University, School of Medicine, Istanbul, Turkey; Department of Anesthesiology and Reanimation, Haseki Research and Training Hospital, Istanbul, Turkey

**Keywords:** Sexual function, Stress urinary incontinence, TOT, FSFI

## Abstract

We evaluate quality of life and sexual function before and after transobturator tape procedure (TOT) using the International Consultation on Incontinence Questionnaire (ICIQ -SF) and Female Sexual Function Index (FSFI). Between 2008 and 2013, 92 patients with stress urinary incontinence (SUI) underwent TOT procedure. A total of 81 patients were sexual active and enrolled in the study. All patients completed the Turkish translation ICIQ -SF and FSFI forms before and 1, 3, 6, 12 months after surgery. To evaluate the impact of incontinence and TOT success on sexual function, we compared patients that were dry after surgery and patients still incontinent and/or facing complication.

All 81 patients completed the study protocol. The total FSFI score was 21.3 ± 7.9 and statistically significant when compare with preoperative total FSFI score (16.2 ± 7.9). The mean postoperative ICIQ -SF score (2 ± 2.9) was also significantly lower than the mean preoperative ICIQ -SF score (17.3 ± 1.8). Complications were encountered in 13 patients, including vaginal erosion (4 patients), de novo urge incontinence (4 patients), vesico-vaginal fistula (1 case), cysto-rectocele (1 case) and high postoperative residue requiring mesh excision (3 patients). Continent (n = 68) patients had a significantly better postoperative total FSFI and ICIQ -SF score against patients who had urine loss.

Our study found a significant improvement of FSFI score and ICIQ -SF score after TOT operation in women with SUI. Additionally, urine loss due to complications was related with worsened FSFI score and ICIQ score compare with healthy patient’s scores.

## Introduction

Stress urinary incontinence (SUI) is a common disorder in the female population and up to 25% of women are faced with deterioration of quality of life due to involuntary urine leakage in their lifespan (Jha et al. 2012). Once transobturator tape (TOT) had been defined for SUI, it quickly became a gold standard surgical procedure due to its minimal invasive nature, high success and acceptable complication rates (Novara et al. 2010; Latthe et al. 2007). Despite the high volume of study addressing the safety and efficiency of TOT, the affect of TOT on female sexual function has yet to be properly assessed.

Female sexual dysfunction (FSD) is a complex issue and defined as a sexual desire, sexual arousal, orgasm and sexual pain disorder that is related with personal distress (Basson et al. 2000). Negative impact of SUI on sexual life was well-defined in previous reports and embarrassment, low desire, arousal difficulty, leakage during intercourse and dysparonia are the most common complaints (Handa et al. 2004). In this perspective, successfully treating SUI may improve sexual function. Whereas surgical complications such as mesh erosion, pain during coitus and de novo urgency contribute sexual dysfunction (Shah et al. 2005; Salonia et al. 2004; Rogers et al. 2004).

The aim of the study was to evaluate the effect of TOT procedure on sexual function and quality of life in sexual active patients using the Female Sexual Function Index (FSFI) and International Consultation on Incontinence Modular Questionnaire (ICIQ).

## Materials and methods

In this prospective study, we collected data on patients undergoing TOT procedure for SUI between 2008 and 2013. Ethical approval was obtained from local ethics committee, and all patients provided written informed consent for participation. Only sexual active patients were enrolled in the study; participants having had at least one sexual activity within 3 months prior to surgery were accepted as sexual active. All patients answered the questionnaires including a validated form of FSFI and ICIQ -SF in Turkish language (Mert & Özen 2011; Çetinel et al. 2004). These questionnaires were applied to patients preoperatively and performed again at the 1, 3, 6, and 12 months postoperatively. Other exclusion criteria were urge incontinence, neurogenic bladder, previous incontinence surgery, severe mental or neurological disorders and refusal to consent.

Demographic characteristics of patients including age, body-mass index, co-morbidities, number of pregnancies and menopausal status were recorded preoperatively. All patients underwent physical examination included caught test and Q-tip test. Urine culture and urinary Ultrasonography for post-voiding residue and dilatation of upper urinary tract was performed. Preoperative urodynamic assessment was performed in accordance with to International Continence Society guidelines (Abrams et al. 2002).

The procedures were performed as described by Delorme (Delorme et al. 2004). Intraoperative and postoperative complication (bladder perforation, hematomas, urethral or vaginal injury, bladder outlet obstruction, infection, mesh erosion and de novo urgency) were recorded. Success was evaluated by history taking, caught test and Q-tip test in follow-ups.

The FSFI is a detailed anonymous questionnaire described by Rosen et al., who demonstrated its validity and credibility (Wiegel et al. 2005). To obtain self-report measurement of female sexual function, 19 questions under 6 domains, including sexual desire (score range 2-10), sexual arousal (score range 0-20), lubrication (score range 0-20), satisfaction (score range 2-15), orgasm (score range 0-15) and pain during sexual intercourse (score range 0-15) was determined and total score was calculated by adding the sum of 6 domains.

The ICIQ-SF is a self-completion questionnaire used to assessment the symptoms and impact of incontinence on quality of life (Avery et al. 2004). The simple scoring system can be easily understood by patients of different socio-economic status and quickly completed with typically little missing data. Previous studies have demonstrated that it accurately measures incontinence and reflects theories associated to incontinence (Kelleher et al. 1997; Sandvik et al. 1993).

### Statistical analyses

The IBM SPSS 21.0 computer software was used during statistical analyses. Data were expressed by means, medians, standard deviations, percentages, minimum and maximum values. Number of parity, ICIQ-SF scores and related results were found to be non-homogenous between patients. Comparisons of preoperative and postoperative FSFI and ICIQ-SF scores were performed by Paired samples t-test and Wilcoxon non-parametric 2 related samples test respectively. Comparisons of FSFI and ICIQ-SF related results between complicated and non-complicated patients were performed by independent samples t-test and Mann Whitney U-test respectively.

## Results

The totally TOT procedure was performed to 92 patients between 2008 and 2013. Eleven patients were excluded from the study due to reasons mentioned above and a total of 81 patients completed the study protocol. The mean age and mean body mass index of 81 patients were 51.6 ± 10.3 years (range from 26 to 79) and 29.7 ± 2.3 kg/m^2^ (range from 23 to 33), respectively. The demographic characteristics of patients are listed in Table 1.

**Table 1:** **Demographic data of all patients**

Number of patients	81	
Mean age (years)	51.6 ± 10.3	(range 26-79)
Mean BMI (kg/m^2^)	29.7 ± 2.3	(range 23-33)
Parity	3.3 ± 1.6/3	(range 1-9)
Mean operation time (minutes)	35 ± 20.6	(range 15-90)
Hypertension	40	49.4%
Diabetes mellitus	15	18.5%
Number of menopause patients	31	38.2%
Mean follow up time (months)	29.8 ± 8.9	(range 19.2 - 38.3)

With the exception of 2 patients, all women who had an active sexual life preoperatively reported sexual activity after TOT procedures. These two patients were excluded from the study. The total FSFI score increased between preoperative assessment and the 12 months postoperative follow up- from 16.2 ± 7.9 (range from 2 to 36) to 21.3 ± 7.9 (range from 2 to 36). Additionally, significant improvements were found in all domains between preoperative and postoperative assessment among sexual desire, sexual arousal, lubrication, orgasm, satisfaction and pain. Furthermore, the mean postoperative ICIQ -SF score was significantly lower than the mean preoperative ICIQ -SF score (an indicator of improvement of quality of life) (Table 2).

**Table 2:** **Preoperative and postoperative FSFI and ICI-Q SF score**

	Preoperative	Postoperative	P
FSFI score	17.4 + 7.4	21.5 + 7.7	
ICI-Q SF score	17.3 + 1.8	2.0 + 2.9	
FSFI score increase	4.1 + 3.8		0.001
ICIQ-SF score decrease	15.5 + 3.3		0.001

At 29.8 (range 19.2 - 38.3) months follow-up after surgery, 68 of 81 patients had no incontinence complaints with a negative caught test and Q type test at physical examination. The remaining 13 patients faced postoperative complications such as vaginal erosion, de novo urge incontinence, urinary retention and vesico-vaginal fistula (Table 3). We matched FSFI sum score of 13 patients with TOT complications to patients without complications. According to preoperative total FSFI and ICIQ -SF score, total FSFI score improvement and ICIQ -SF score reductions were only found in patients without complications (Table 4).

**Table 3:** **Complications and performed solutions**

Complication	Number	Solution
Vaginal erosion	4	Mesh excision
Denovo urge incontinence	4	Ant cholinergic treatment
Fail due to unrecognized vesico-vaginal fistula	1	Vesco-vaginal fistula repair
Urinary retension + high post operative residue	3	Mesh excision

**Table 4:** **Differences between complicated and non complicated cases according to FSFI and ICIQ-SF scores**

	Complicated cases	Non complicated cases	p value
Number	13	68	
Preoperative FSFI score	15.8 + 6.2	17.7 + 7.5	0.404
Preoperative ICI-Q SF score	16.9 + 1.3	17.3 + 1.7	0.239
Postoperative FSFI score	16.4 + 5.6	22.1 + 8.3	0.022
Postoperative ICI-Q SF score	4.4 + 4.8	1.2 + 1.3	0.01
FSFI score increase	0.6 + 2.3	4.9 + 3.6	0.001
ICIQ-SF score decrease	12.9 + 5.2	16.1 + 2.2	0.033

## Discussion

Stress urinary incontinence (SUI) is defined as the involuntary leakage of urine due to increased abdominal pressure without detrusor muscle contraction. Since its introduction to the field of urology by Delorme, TOT achieved by placing the polypropylene mesh between two obturator foremen to suspend urethra has become a common procedure for treating SUI (Poza et al. 2008; Giberti et al. 2007).

Recently, female sexuality has become the focus of attention as an important part of women’s health and is accepted as a basic human right according to World Health Organization (World Health Organization 2002). Female sexual dysfunction may arise from anatomical or neurological disorders, medication, alcohol abuse or cultural factors. Female sexual dysfunction is more common in women with pelvic floor disorders and urinary incontinence when compare with the healthy female population (Kingsberg & Janata 2007; Basson & Schultz 2007). Additionally, life period, stress incontinence, aging, menopausal status and presence of co-morbidities are associated with deteriorated sexual function (Davison et al. 2008).

Despite numerous investigations addressing the effect of SUI surgery on sexual function, results have been heterogeneous (Mazouni et al. 2004; Pace & Vicentini 2008). Although some studies reported improved function, others reported conflicting results with deterioration or no change in symptoms. One of reason (e.g., study design including varying age, hormonal status, co-morbidities, heterogeneity of coexistence of pelvic organ prolepses, difference evaluation criteria on success and retrospective design of studies) has been shown to account for these variable results (Naumann et al. 2013).

Amelioration of urine leakage during intercourse and regaining self-confidence are believed to be principal reasons for recovery of sexual function. In our study, according to the FSFI patients showed a significant improvement after TOT. The mean preoperative total FSFI was increased from 16.2 ± 7.9 to 21.3 ± 7.9 at the 12 month follow-up (p < 0,01). All FSFI sub domains changed positively during the same period. Contrary to our study, Kim et al. have showed that there were no significant differences in the FSFI domain scores before and after SUI surgery. (Kim & Choi 2008).

In contrast, other studies have demonstrated impairment of sexual function after TOT. Specifically, incision of anterior vaginal wall and damage to vascular and neuronal tissue of the vagina and clitoris may be associated arousal and orgasmic dysfunction (Katz 2003). In addition, vaginal scarring, erosion of sling, dysparonia and partner discomfort can contribute to sexual dysfunction (Dragisic & Milad 2004). We believe that complications and incontinence after TOT are the main factors for deterioration of sexual function. Complications were encountered in 13 patients leading to urine loss. These patients reported a worsened total FSFI score in comparison to continent patients.

Menopausal status is an important condition affecting quality of life in women (Fernandes et al. 2014). However, according to Berra et al., preoperative and postoperative evaluation of sexual function in premenopausal and menopausal women identified no statistically differences. Additionally, Berra et al. demonstrated that sexual dysfunction was associated with more personal distress in premenopausal women (Berra et al. 2010). In our study, total preoperative and postoperative FSFI scores were significantly better in premenopausal women relative to menopausal women. However, the improvement in total FSFI score after surgery was not significantly different between the premenopausal and menopausal groups.

Small sample size was the major limitation in this study. Secondly, we investigated only impact of TOT procedure on female sexual function. We believe that comparative studies addressing different incontinence surgery techniques may be better able to reveal the impact of incontinence surgery on female sexual function. Additionally, the FSFI questionnaire does not query incontinence during intercourse.

## Conclusions

Although female sexual dysfunction is a difficult and complex issue, it is clear that urine incontinence negatively impacts sexual function. Our study demonstrated that the TOT procedure lead to significant improvement in sexual function and quality of life in mid-term follow-up.

## References

[CR1] Abrams P, Cardozo L, Fall M, Griffiths D, Rosier P, Ulmsten U, Van Kerrebroeck P, Victor A, Wein A (2002). The standardisation of terminology of lower urinary tract function: report from the Standardisation Sub-committee of the International Continence Society. Neurourol Urodyn.

[CR2] Avery K, Donovan J, Peters TJ, Shaw C, Gotoh M, Abrams P (2004). ICIQ: A brief and robust measure for evaluating the symptoms and impact of urinary incontinence. Neurourol Urodyn.

[CR3] Basson R, Schultz WW (2007). Sexual sequelae of general medical disorders. Lancet.

[CR4] Basson R, Berman J, Burnett A, Derogatis L, Ferguson D, Fourcroy J, Goldstein I, Graziottin A, Heiman J, Laan E, Leiblum S, Padma-Nathan H, Rosen R, Segraves K, Segraves RT, Shabsigh R, Sipski M, Wagner G, Whipple B (2000). Report of the international consensus development on female sexual dysfunction: Definitions and classifications. J Urol.

[CR5] Berra M, De Musso F, Matteucci C (2010). The impairment of sexual function is less distressing for menopausal than for premenopausal women. J Sex Med.

[CR6] Çetinel B, Oscan B, Can G (2004). The validation study of ICIQ-SF Turkish version. Turk J Urol.

[CR7] Davison SL, Bell RJ, LaChina M, Holden SL, Davis SR (2008). Sexual function in well women: Stratification by sexual satisfaction, hormone use, and menopause status. J Sex Med.

[CR8] Delorme E, Droupy S, De Tayrac R, Delmas V (2004). Transobturator tape (Uratape): a new minimally-invaziv procedure to treat female urinary incontinence. Eur Urol.

[CR9] Dragisic KG, Milad MP (2004). Sexual functioning and patient expectations of sexual functioning after hysterectomy. Am J Obstet Gynecol.

[CR10] Fernandes T, Costa-Paiva LH, Pinto-Neto AM (2014). Efficacy of vaginally applied estrogen, testosterone, or polyacrylic acid on sexual function in postmenopausal women: a randomized controlled trial. J Sex Med.

[CR11] Giberti C, Gallo F, Cortese P, Schenone M (2007). Transobturator tape for treatment of female stress urinary incontinence: objective and subjective results after a mean follow-up of two years. Urology.

[CR12] Handa VL, Harvey L, Cundiff GW, Siddique SA, Kjerulff KH (2004). Sexual function among women with urinary incontinence and pelvic organ prolapse. Am J Obstet Gynecol.

[CR13] Jha S, Ammenbal M, Metwally M (2012). Impact of incontinence surgery on sexual function: a systematic review and meta-analysis. J Sex Med.

[CR14] Katz A (2003). Sexuality after hysterectomy: a review of the literature and discussion of nurses’ role. J Adv Nurs.

[CR15] Kelleher C, Cardozo L, Khullar V, Salvatore S (1997). A new questionnaire to assess the quality of life of urinary incontinent women. Br J Obstet Gynaecol.

[CR16] Kim DY, Choi JD (2008). Change of sexual function after midurethral sling procedure for stress urinary incontinence. Int J Urol.

[CR17] Kingsberg SA, Janata JW (2007). Female sexual disorders: Assessment, diagnosis, and treatment. Urol Clin North Am.

[CR18] Latthe PM, Foon R, Toozs-Hobson P (2007). Transobturator and retropubic tape procedures in stres urinary incontinence: asystemic review and meta-analysis of effectiveness ond complications. BJOG.

[CR19] Mazouni C, Karsenty G, Bretelle B, Bladou F, Gamerre M, Serment G (2004). Urinary complications and sexual function after the tension-free vaginal tape procedure. Acta Obstet Gynecol Scand.

[CR20] Mert DG, Özen NE (2011). Genel psikiyatri polikliniğine başvuran kadın hastalarda cinsel işlev bozukluğu ve ilişkili sosyokültürel parametrelerin değerlendirilmesi. Klinik Psikiyatri.

[CR21] Naumann G, Steetskamp J, Meyer M, Laterza R, Skala C, Albrich S, Koelbl H (2013). Changes in sexual function and quality of life after single-incision mid-urethral sling for treatment of female stress urinary incontinence. Eur J Obstet Gynecol Reprod Biol.

[CR22] Novara G, Artibani W, Barber MD, Chapple CR, Costantini E, Ficarra V, Hilton P, Nilsson CG, Waltregny D (2010). Updated systematic review and meta-analysis of the comparative data on colposuspensions, pubovaginal slings, and midurethral tapes in the surgical treatment of female stress urinary incontinence. Eur Urol.

[CR23] Pace G, Vicentini C (2008). Female sexual function evaluation of the tension-free vaginal tape (TVT) and trans-obturator suburethral tape (TOT) incontinence surgery. Results of a prospective study. J Sex Med.

[CR24] Poza JL, Pal F, Sabadell J, Sánchez-Iglesias JL, Martínez-Gómez X, Xercavins J (2008). Trans-obturator suburethral tape for female stress incontinence: a cohort of 254 women with 1-year to 2-year follow-up. Acta Obstet Gynecol Scand.

[CR25] Rogers RG, Kammerer-Doak D, Darrow A, Murray K, Olsen A, Barber M, Qualls C (2004). Sexual function after surgery for stress urinary incontinence and or pelvic organ prolapse: a multicenter prospective study. Am J Obstet Gynecol.

[CR26] Salonia A, Zanni G, Nappi RE, Briganti A, Dehò F, Fabbri F, Colombo R, Guazzoni G, Di Girolamo V, Rigatti P, Montorsi F (2004). Sexual dysfunction is common in women with lower urinary tract symptoms and urinary incontinence: results of a cross-sectional study. Eur Urol.

[CR27] Sandvik H, Hunskaar S, Seim A, Hermstad R, Vanvik A, Bratt H (1993). Validation of a severity index in female urinary incontinence and its implementation in an epidemiological survey. J Epidemiol CommunityHealth.

[CR28] Shah SM, Bukkapatnam R, Rodríguez LV (2005). Impact of vaginal surgery for stress urinary incontinence on female sexual function: is the use of polypropylene mesh detrimental?. Urology.

[CR29] Wiegel M, Meston C, Rosen R (2005). The female sexual function index (FSFI): Cross-validation and development of clinical cut-off scores. J Sex Marital Ther.

[CR30] World Health Organization (2002). Sexual health: Working definitions.

